# Toe Walking Secondary to Tethered Spinal Cord Caused by Ganglioneuroma: A Case Report

**DOI:** 10.7759/cureus.73035

**Published:** 2024-11-05

**Authors:** Athkar H Alhajjaj, Mohammed A Almudayfir, Wafa S Alotaibi, Deem M AL-Sedais, Mohammed A Alotaibi

**Affiliations:** 1 Orthopedic Surgery, Prince Sultan Military Medical City, Riyadh, SAU; 2 Medicine, King Saud University, Riyadh, SAU

**Keywords:** case report, posterior mediastinum mass, tethered spinal cord, tip toeing, toe walking

## Abstract

Toe walking is described by the incapacity to achieve heel strike during the stance phase of the gait cycle. When a child presents with toe walking beyond three years of age, a thorough evaluation is crucial. We report a case of a seven-year-old female presented with painless toe walking that started two years prior to presentation. Medical history and physical examination were unremarkable. Various investigations were done to rule out the central causes of tip-toeing. MRI revealed a 50x76x62 mm lesion within the posterior mediastinum extending through the left neural foramina of T6 that caused right-sided spinal deviation without spinal cord compression. MRI findings were suggestive of a tethered spinal cord secondary to ganglioneuroma. The tumor was excised surgically, and the patient underwent bilateral Achilles tendon lengthening using the hoke technique. Three months after surgery, with the help of physical therapy, both feet were in plantigrade, and the deformity was resolved. Moreover, the patient walked with normal gait, alignment, and full range of motion. Toe walking in children under three years of age is often viewed as a normal gait variant during the time of independent locomotion. However, it is important to rule out underlying causes when it persists beyond three years of age.

## Introduction

Toe walking is described by the incapacity to achieve heel strike during the stance phase of the gait cycle. It is commonly manifested during the time of independent walking and is often viewed as a normal variation of gait development in children younger than three years of age [1,2]. When it persists beyond three years of age, it is considered an abnormal gait type [3]. Toe walking is commonly associated with medical conditions such as autism spectrum disorders (ASDs), cerebral palsy, muscular dystrophies, and global developmental delay [2]. However, it could stem from structural problems like a contracted tendon or compensation for a short limb. In instances where a child demonstrates toe walking without an underlying medical etiology beyond three years of age, a diagnosis of idiopathic toe walking is made [4]. The prevalence of idiopathic toe walking in children is estimated to range between 2% and 12% [3]. It is important to thoroughly evaluate a child with toe walking, including medical history (prenatal, intrapartum, and postnatal), gait evaluation, musculoskeletal examination, and neurologic examination [1,3]. We report an interesting case of toe walking in a seven-year-old girl presented with toe walking secondary to a tethered spinal cord.

## Case presentation

We present a seven-year-old female, medically free, a product of cesarean section with no history of intensive care admissions, vaccination up to date, and normal developmental milestones. The patient was initially brought to the orthopedic clinic by her parents, complaining of a tip-toe walking gait that started two years before the presentation.

The patient's chief complaint was a painless tip-toeing gait that started suddenly two years ago, without a history of pain, tripping, or falling. She did not have constitutional symptoms nor a family history of similar issues, and her past medical and surgical history was unremarkable.

**Figure 1:**
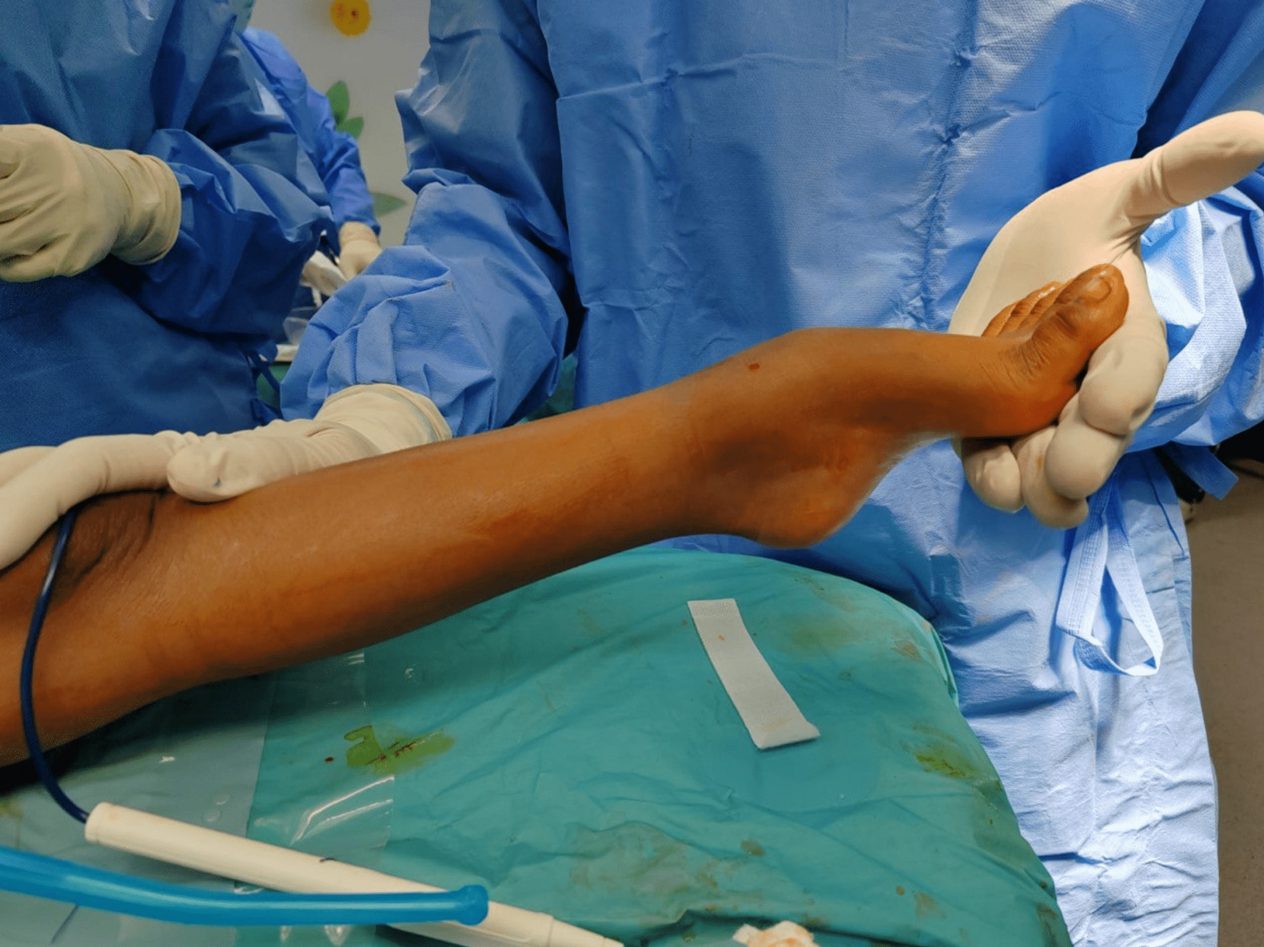
Preoperative clinical picture of the left ankle showing equinus deformity

**Figure 2:**
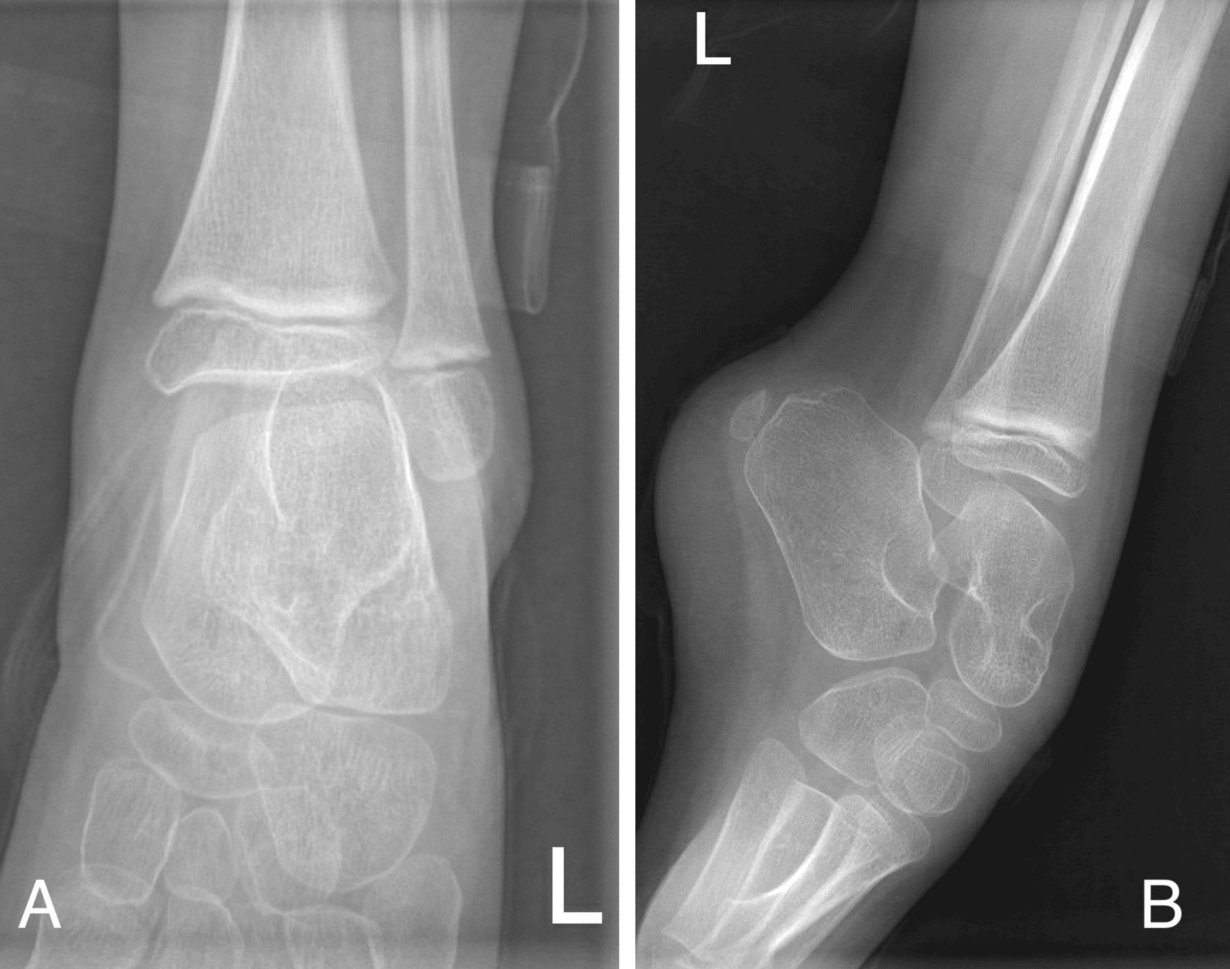
Preoperative X-ray of the left side Preoperative anterior-posterior (A) and lateral (B) X-ray of left ankle showing equinus deformity without obvious bony abnormality.

**Figure 3:**
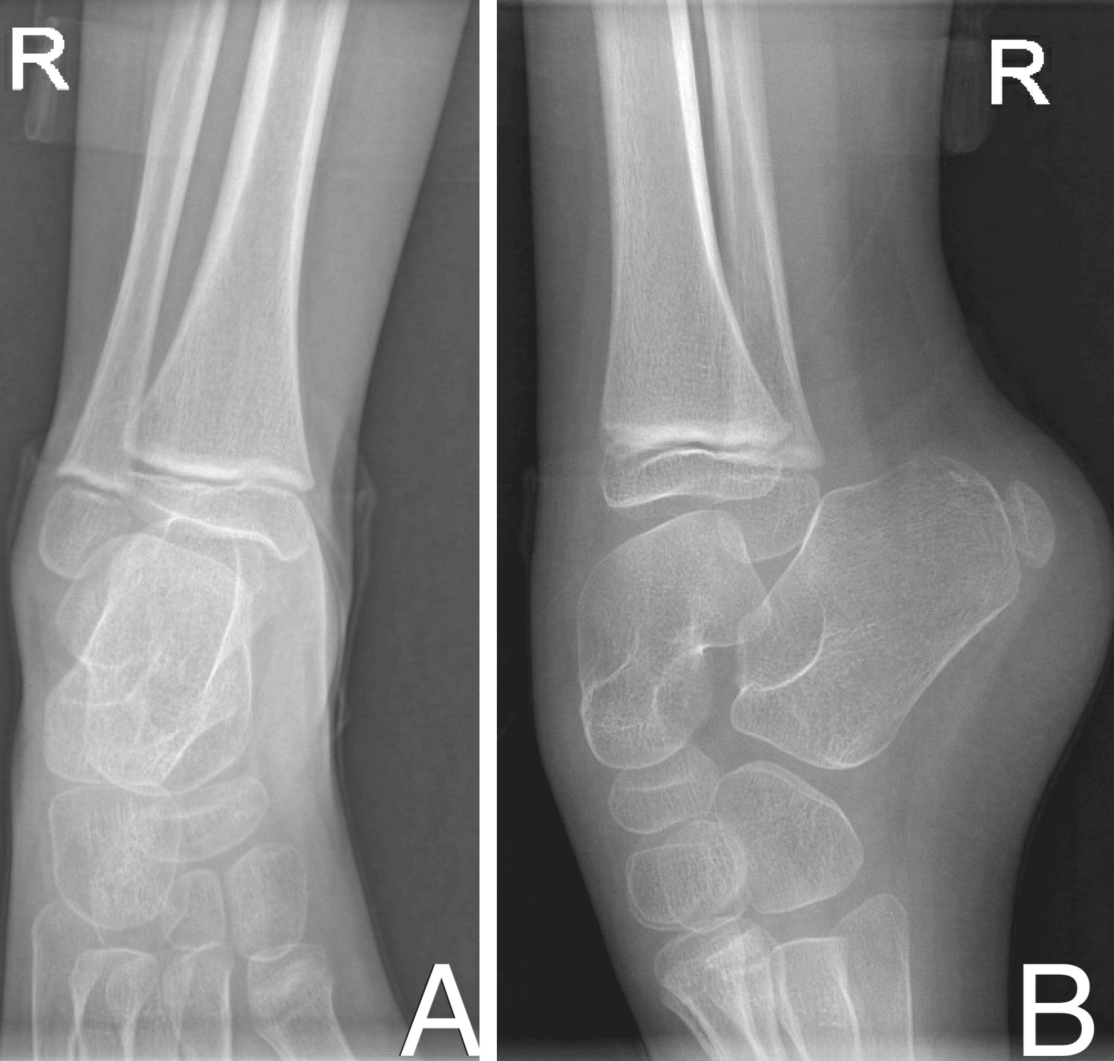
Preoperative X-ray of the right side Preoperative anterior-posterior (A) and lateral (B) X-ray of right ankle showing equinus deformity without obvious bony abnormality.

Physical examination showed tip-toeing walking gait and equinovarus feet bilaterally (Figure 1). There was no muscle atrophy or other deformity. Range of motion exam revealed full planter flexion with limited dorsiflexion, full inversion, and eversion. The patient also had a bilateral tight Achilles tendon and a negative Silfverskiöld test, which indicated the patient's normal gastrocnemius muscle. Muscle power was normal, and the Gower sign was absent. Her neurovascular examination was normal, and the initial bilateral X-rays showed equinus deformity without obvious bony abnormality (Figures 1-3). 

**Figure 4:**
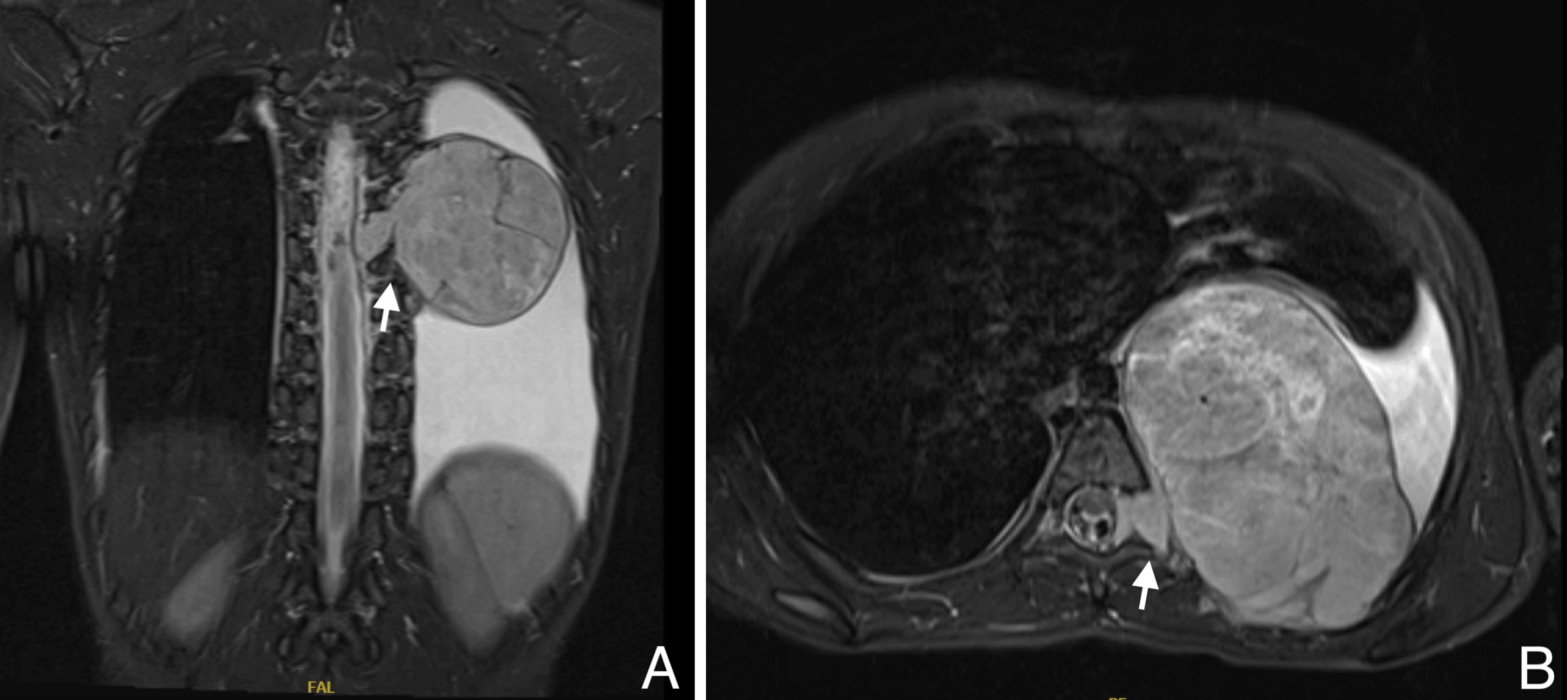
T2 MRI of the chest Preoperative T2 image MRI of the chest, coronal (A), and axial (B) views showing a 50x76x62 mm lesion within the posterior mediastinum extending through the left neural foramina of T6 that caused right-sided spinal deviation without spinal cord compression.

Medical workups in the form of laboratory investigations and X-ray imaging were conducted to identify the cause of tip-toeing. Investigation revealed a normal metabolic profile. However, MRI images revealed a 50x76x62 mm lesion within the posterior mediastinum extending through the left neural foramina of T6 that caused right-sided spinal deviation without spinal cord compression (Figure 4). MRI findings were most likely referring to a neurogenic tumor as in ganglioneuroblastoma.

A multidisciplinary meeting was conducted, and the decision was made to proceed with tumor excision. The patient was taken to the operating room under the combined effort of the pediatric surgery and neurosurgery team, and the tumor was excised successfully without complication. The pathology report revealed mature ganglioneuroma with no evidence of malignancy.

**Figure 5:**
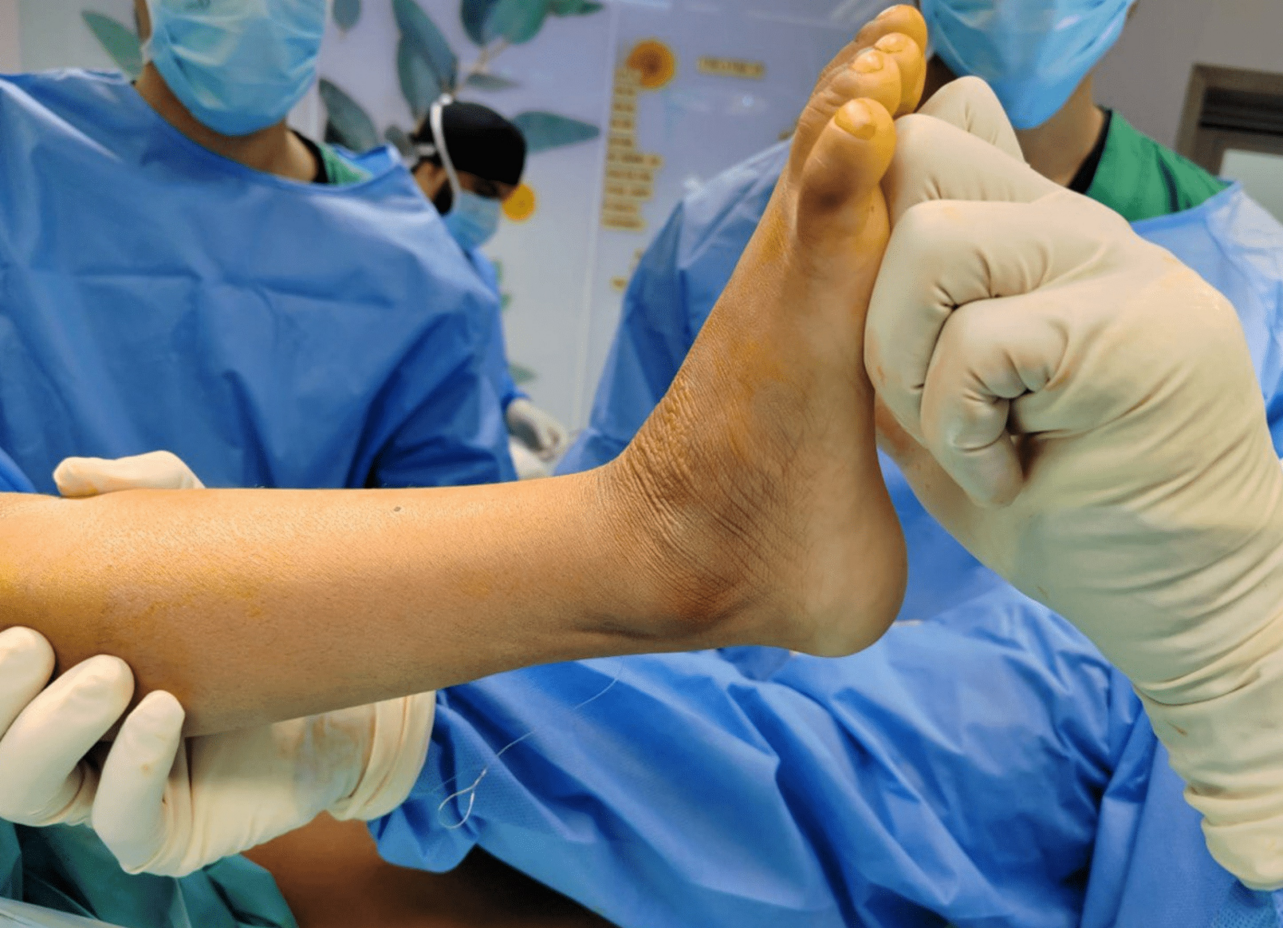
Immediate postoperative clinical picture

Three months after the tumor excision, the patient completely recovered; however, she still suffered from tip-toeing, which was her initial complaint. Conservative treatment of her tip-toeing was not successful, so surgery was discussed with the patient's parents, and the decision was made to proceed with surgery. The patient underwent bilateral Achilles tendon lengthening using the hoke technique with three consecutive incomplete tenotomies of the Achilles tendon to release the tightness, plantar fascia release, and percutaneous flexors release. Intraoperative dorsiflexion was satisfactory (Figure 5), and a full cast above the knee was applied. Surgery was uneventful, and the patient shifted to recovery in very good condition. 

The patient was followed regularly in our clinic. Her surgical scars healed nicely. The above knee cast was kept for six weeks. After cast removal, ankle foot orthosis was applied, and the patient started physical therapy with gait training.

**Figure 6:**
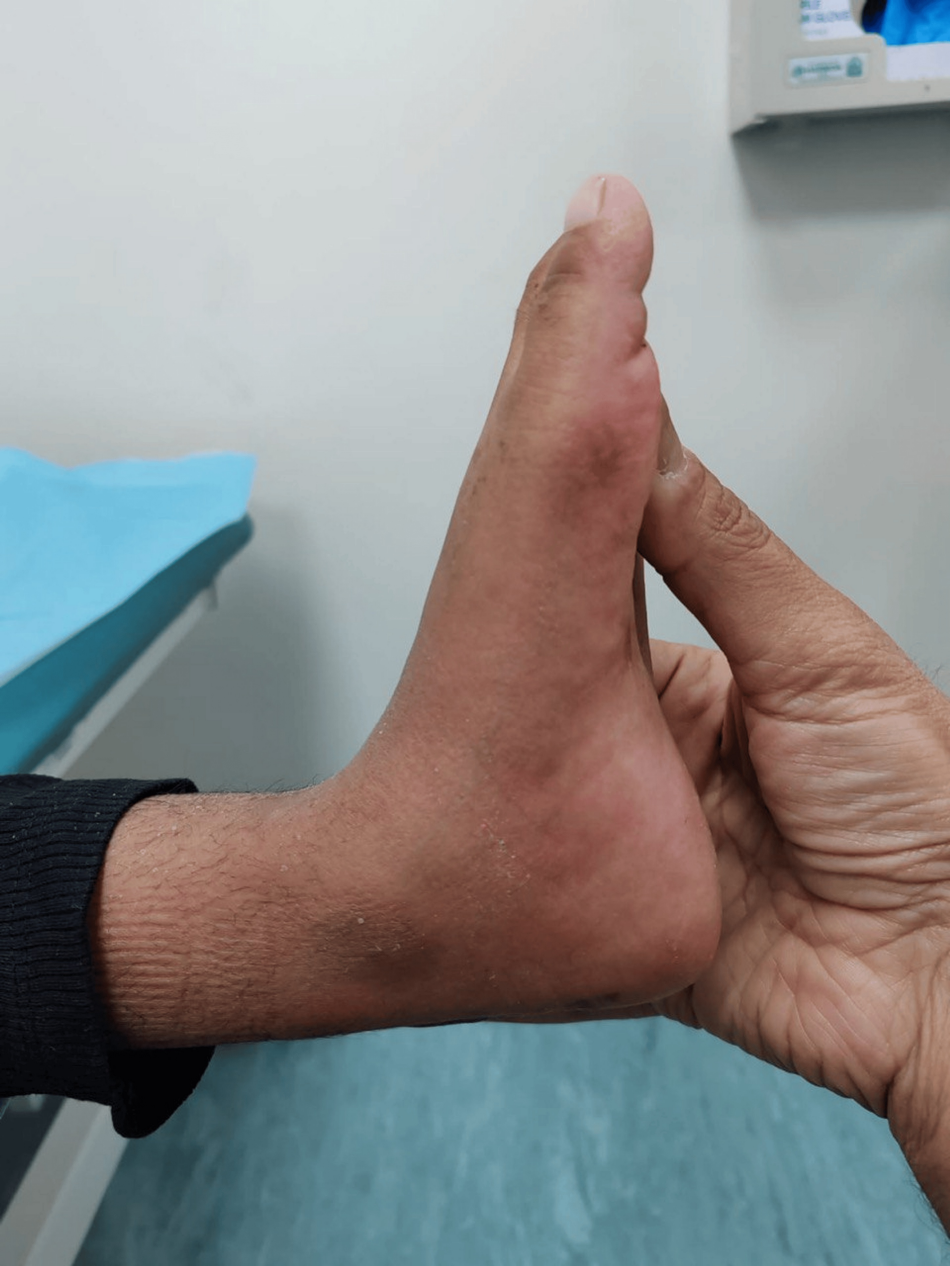
Three months postoperatively Clinical picture three months after surgery showing normal ankle alignment and neutral position.

**Figure 7:**
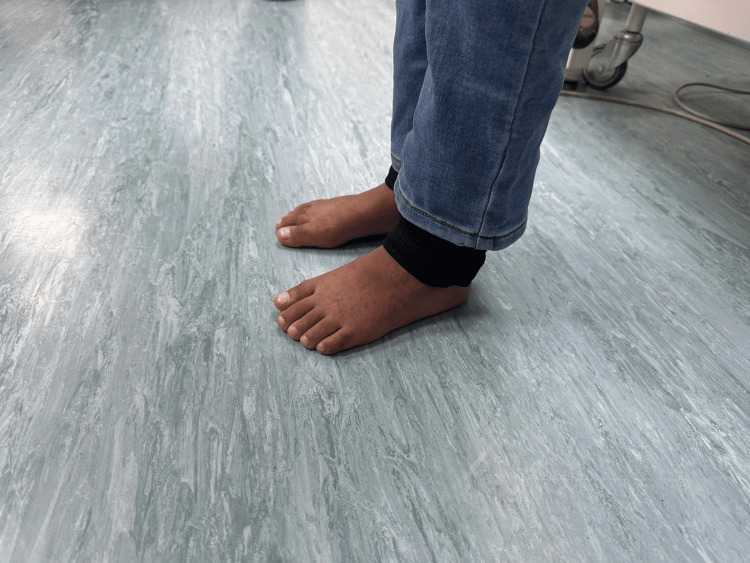
Three months postoperatively Three months after surgery showing normal standing posture of bilateral feet.

**Figure 8:**
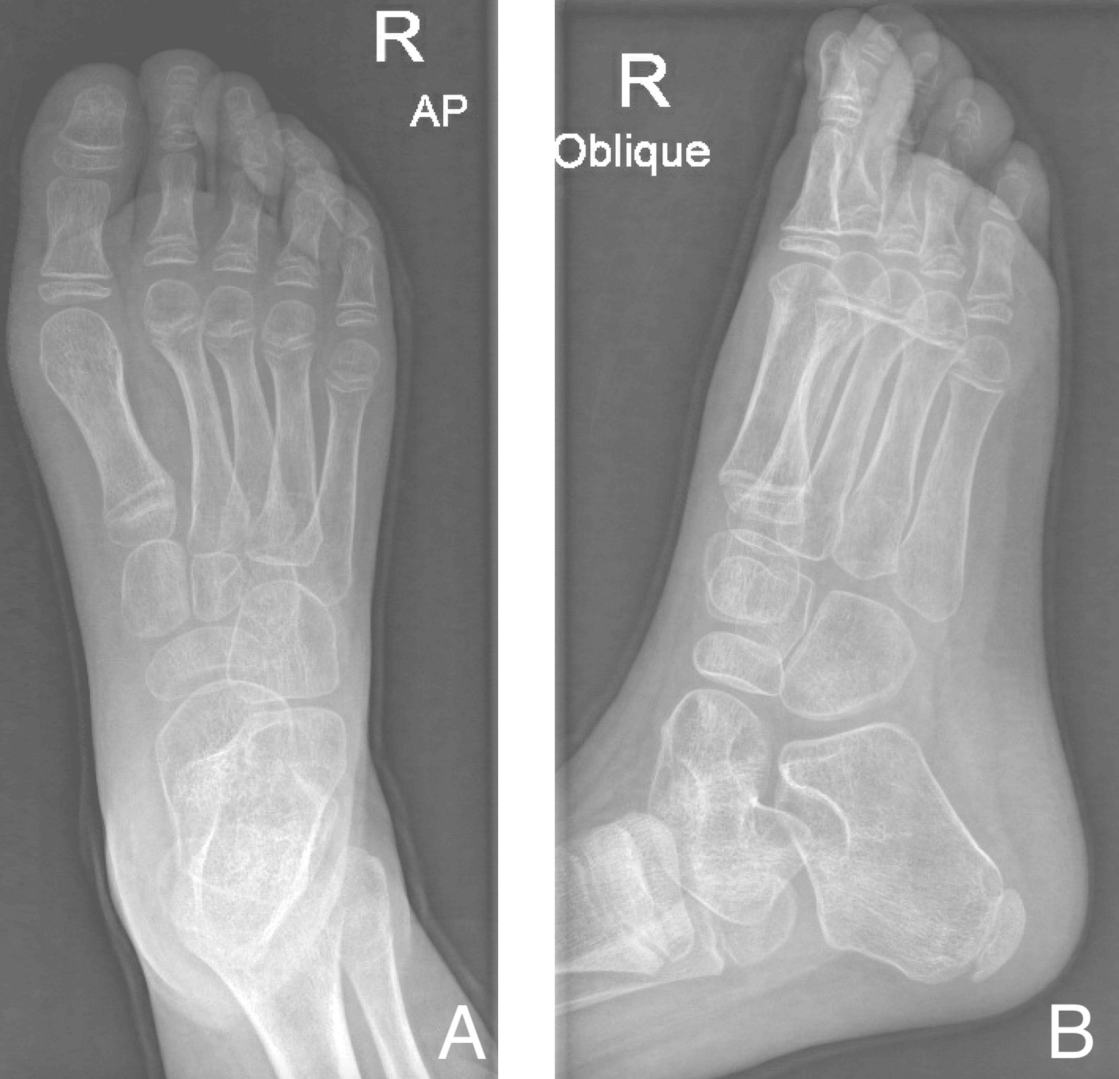
Postoperative X-ray of the right ankle Postoperative anterior-posterior (A) and oblique (B) X-ray of right ankle showing normal ankle alignment.

**Figure 9:**
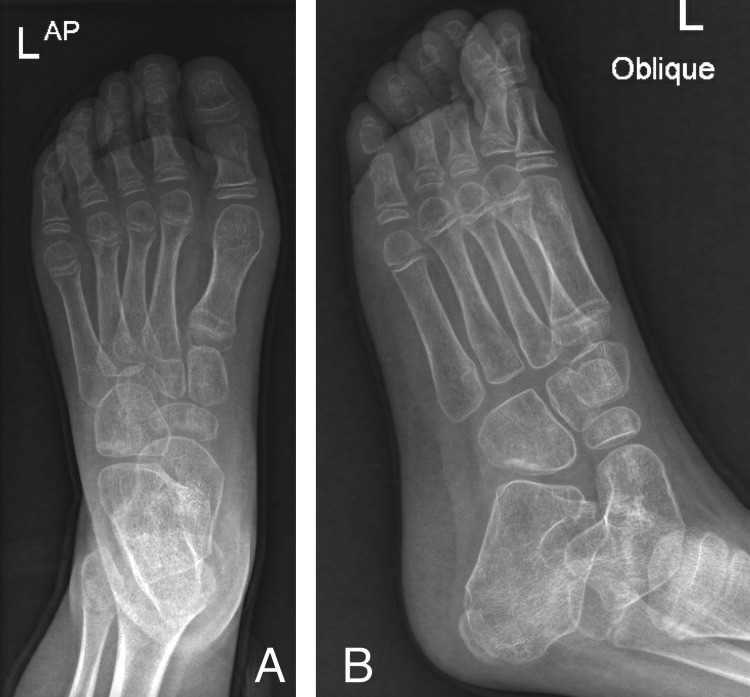
Postoperative X-ray of the left side Postoperative anterior-posterior (A) and oblique (B) X-ray of left ankle showing normal ankle alignment.

Three months following the surgery, the patient was compliant with physical therapy, with a bilateral foot in plantigrade, healed scars, and improved deformity (Figures 6-7). The patient walked with normal gait and alignment, full range of motion, and intact distal neurovascular examination. Moreover, postoperative X-ray revealed normal alignment (Figures 8-9), and both patient and parents were happy with the outcome. 

## Discussion

Tip-toeing gait is a common presentation in pediatric orthopedic practice and is usually a concern for parents. Therefore, it's crucial for practicing pediatric orthopedic surgeons to be familiar with its natural history and its management. Tip-toeing gait basically means the absence of heel strike during the stance phase of the gait cycle. It's important to recognize that a mild form of tip-toeing under the age of three years of age is considered a normal benign finding [1,2]. Severe forms of tip-toeing in patients older than three years warrant medical care and orthopedic surgeon assessment. Tip-toeing is most commonly idiopathic; however, other causes must be considered while managing a child with tip-toeing. These causes include cerebral palsy, muscular dystrophy, neuromuscular disorders, and autism [2]. Even though idiopathic tip-toeing is the most common case presentation, it's a diagnosis of exclusion, and other secondary causes must be considered and excluded. A thorough history and physical examination are essential cornerstones in approaching these cases [3,5], but further work is usually recommended and guided by history and physical examination. Laboratory testing, such as creatine phosphokinase, is a useful tool for screening muscular diseases and myopathy, and in some cases, muscle biopsy is needed for accurate diagnosis. Formal gait analysis should be utilized, and it helps to differentiate cases of cerebral palsy and other neuromuscular disorders and idiopathic toe walking [2]. Nerve conduction studies and electromyography help physicians differentiate between neurological cases and other pathologies [6]. Brain and whole spine MRIs are a helpful tool to assess central causes and rule out tethering of the spinal cord [7]. Furthermore, spinal MRI should be considered in patients with late tip-toeing gait, especially if the patient had a normal gait at a younger age [8]. 

Management of tip-toeing depends on the patient's age, associated conditions, and severity of the disease. It is essential to diagnose and manage pathological causes of tip-toeing gait properly, and it should be treated only after managing or excluding secondary causes. Young patients with mild disease are usually treated with conservative treatment that includes stretching exercises, night splinting, and serial casting, which is usually sufficient. Ankle-foot orthosis (AFO) is a useful tool that also helps patients. Surgical lengthening should be considered in older children with severe deformity and those who fail to improve with conservative treatment [9]. Surgical Achilles lengthening can be done either percutaneously or open, and the decision is guided by patient characteristics and surgeon preference. Moreover, patients who undergo surgical lengthening need to be immobilized with a cast for six weeks after surgery, and after that, physiotherapy should be started; ankle foot orthosis use during the first three months after surgery is recommended to prevent recurrence. Anyhow, the postoperative occurrence of toe walking might happen [10-13].

## Conclusions

We reported a case of a late presentation of toe walking gait in a healthy young girl, and an investigation revealed a tethered spinal cord secondary to ganglioneuroma. She underwent tumor excision. After full recovery, she underwent Achilles tendon release. The patient tolerated surgery well and achieved full recovery during her follow-ups. In conclusion, toe walking gait is a common complaint in pediatric orthopedics; detailed history and physical examinations will guide treating physicians toward specific differentials. Moreover, a full workup is recommended to rule out secondary causes. Physicians should always have a high index of suspicion for underlying central causes of toe walking that can be easily missed due to the lack of systemic manifestations that present with toe walking. Furthermore, management can vary depending on factors such as severity, the presence of medical causes, and individual patient considerations.
